# Development of an Online Genome Sequence Comparison Resource for *Bacillus cereus sensu lato* Strains Using the Efficient Composition Vector Method

**DOI:** 10.3390/toxins15060393

**Published:** 2023-06-12

**Authors:** Kui Wang, Changlong Shu, Alejandra Bravo, Mario Soberón, Hongjun Zhang, Neil Crickmore, Jie Zhang

**Affiliations:** 1 State Key Laboratory for Biology of Plant Diseases and Insect Pests, Institute of Plant Protection, Chinese Academy of Agricultural Sciences, Beijing 100193, China; 2 School of Plant Protection, Anhui Agricultural University, Hefei 230036, China; 3 Departamento de Microbiología Molecular, Instituto de Biotecnología, Universidad Nacional Autónoma de México, Cuernavaca 62250, Mexico; 4 Institute for the Control of Agrochemicals, Ministry of Agriculture and Rural Affairs, Beijing 100125, China; 5 School of Life Sciences, University of Sussex, Brighton BN1 9QG, UK

**Keywords:** *Bacillus thuringiensis*, *B. cereus sensu stricto*, *B. anthracis*, phylogenetic analysis, composition vector tree

## Abstract

An automated method was developed for differentiating closely related *B. cereus sensu lato* (*s.l.*) species, especially biopesticide *Bacillus thuringiensis,* from other human pathogens, *B. anthracis* and *B. cereus sensu stricto* (*s.s.*). In the current research, four typing methods were initially compared, including multi-locus sequence typing (MLST), single-copy core genes phylogenetic analysis (SCCGPA), dispensable genes content pattern analysis (DGCPA) and composition vector tree (CVTree), to analyze the genomic variability of 23 *B. thuringiensis* strains from *aizawai*, *kurstaki*, *israelensis*, *thuringiensis* and *morrisoni* serovars. The CVTree method was the best option to be used for typing *B. thuringiensis* strains since it proved to be the fastest method, whilst giving high-resolution data about the strains. In addition, CVTree agrees well with ANI-based method, revealing the relationship between *B. thuringiensis* and other *B. cereus s.l.* species. Based on these data, an online genome sequence comparison resource was built for *Bacillus* strains called the *Bacillus* Typing Bioinformatics Database to facilitate strain identification and characterization.

## 1. Introduction

The *Bacillus cereus* group, which is also known as *B. cereus sensu lato* (*s.l.*), is a subdivision of the genus *Bacillus*. Currently the *B. cereus* group contains 22 closely related pathogenic and non-pathogenic species [1]. The pathogenic status of *B. cereus* group members is not homogeneous as they contain strains used as probiotics in animal nutrition, such as *B. toyonensis* [2], and also the deadly dangerous pathogen, *B. anthracis*, the virulent pathogen of mammals and causative agent of anthrax [3]. *B. thuringiensis* has been used successfully as an important biological insecticide due to the pesticidal proteins produced by these bacteria, which are toxic against different insect orders, such as Lepidoptera, Coleoptera and Diptera [4,5]. *B. cereus sensu stricto* (*s.s.*) is an opportunistic pathogen that can poison human food [6]. *B. cytotoxycus* is also a pathogenic species that was responsible for a severe foodborne outbreak [7]. On the other hand, the majority of the remaining members of the *B. cereus* group are mostly non-pathogenic species that are not or are rarely associated with illnesses in humans or other animals, such as *B. mycoides* or *B. weihenstephanensis*. The significant impact on human health, agriculture, and the food industry shown by *B. cereus* group members supports the need for an efficient typing and classification system of isolates within the group.

Various typing methods have been used for the identification and taxonomic characterization of *B. cereus s.l.* species, including traditional phenotypic tests, such as flagellar H serotyping [8] and analyzing the presence or absence of virulence plasmids [9], colonial morphology [10], and psychrophilic and thermotolerant abilities [11], as well as genotyping methods, including the analysis of single genes (16S rDNA, *gyrB* and *plcR*) [12,13], amplified fragment length polymorphisms (AFLP) [14], and multi-locus sequence typing (MLST) based on five to seven housekeeping gene sequences [15]. Although these methods provide insights into the genetic diversity and phylogenetic relationships among species classified as *B. cereus s.l.*, problems still remain in distinguishing between organisms that are capable of causing illness or death from those used in industries [16,17]. For example, the biopesticide, *B. thuringiensis,* and human pathogens, *B. anthracis* and *B. cereus s.s.,* are mainly differentiated on the basis of their production of different virulence factors, whose genes are located predominantly on plasmids that can be easily transferred or lost [18]. Furthermore, various studies also have demonstrated that these three species are closely genetically related, which has led to the proposal that they should be recognized as a single species [19,20]. Although *B. thuringiensis*-based products have been considered as safe insecticides [21], the ability to discriminate *B. thuringiensis* from the human pathogenic *B. anthracis* and *B. cereus s.s.* species is essential for the registration of *B. thuringiensis* biological control agents with a Generally Regarded as Safe (GRAS) status.

With the rapid development of genome sequencing technology, bacterial genome sequencing has become a convenient and feasible analysis technique. Thus, strain typing methods based on whole-genome sequences have become more attractive, such as those based on average nucleotide identity (ANI) [22] and digital DNA–DNA hybridization (dDDH) [23]. However, for both methods, the similarity between *B. thuringiensis-* and *B. cereus s.s.*-type strains is greater than the species boundary cutoffs [24]. Carroll et al. found that the current species definitions of this group led to overlapping genomospecies clusters at the conventional 95 ANI genomospecies threshold and proposed a nomenclatural framework, which accounted for genomospecies using resolvable genomospecies clusters obtained at ≈92.5 ANI [25]. According to the framework, the *B. cereus* group consists of eight published genomospecies (*B. cereus s.s.*, *B. mosaicus*, *B. mycoides*, *B. pseudomycoides*, *B. paramycoides*, *B. toyonensis*, *B. cytotoxicus* and *B. luti*), four previously proposed genomospecies (“*B. bingmayongensis*”, “*B. gaemokensis*”, “*B. manliponensis*” and “*B. clarus*”), and six new ones, referred to as “Unknown *B. cereus* group Species 13–18”. The former species, *B. cereus s.s.* and *B. thuringiensis,* were assigned to the *B. cereus s.s.* genomospecies. *B. anthracis* were assigned to the *B. mosaicus* genomospecies, with six other former species *B. albus*, *B. mobilis*, *B. pacificus*, *B. paranthracis*, *B. tropicus* and *B. wiedmannii*. Additionally, *B. mycoides*, *B. weihenstephanensis*, *B. proteolyticus*, and *B. nitratireducens* were assigned to the *B. mycoides* genomospecies. The author also released software BTyper3 (version 3.1.1; Structural and Computational Biology Unit, European Molecular Biology Laboratory, Heidelberg, Germany; available from https://github.com/lmc297/BTyper3, accessed on 13 March 2021), which can be used to assign *B. cereus s.l.* isolates to taxonomic units within this proposed framework. However, this tool requires complex computing resources and some programming ability, which means that it may not be user-friendly for fast-paced and large-scale data analysis by microbiologists who are unfamiliar with the Linux command line environment. Developing an automated and low-resource-consuming system is essential for differentiating between closely related *B. cereus s.l.* isolates.

Composition vector tree (CVTree) analysis is an alignment-free method based on subsequences of a defined length (named k-strings). Using the CVTree algorithm, the genome sequences are cut into small k-strings, and each organism is represented by a composition vector (CV), which is calculated using the difference between the frequencies of k-strings and the prediction frequencies via the Markov model. Then, the similarity between the two genomes is measured according to the cosine of the angle between two CVs [26]. Finally, dissimilarity matrices are generated for phylogenetic studies. This CVTree analysis has been effectively applied to several phylogenetic studies of different datasets, including archaea [27], prokaryotes [28], fungi [29], chloroplast [30] and DNA viruses [31]. However, this method has not been previously applied to *B. cereus s.l.* group strains. The aim of this study was to find the most suitable method for differentiating between strains within the *B. cereus* group, with a particular emphasis on *B. thuringiensis* subspecies/genomospecies. The method of achieving the differentiation of *B. thuringiensis* strains into serovars was developed on the basis of flagellar antigens by de Barjac and Bonnefoi [32], and it has been the main strategy for typing *B. thuringiensis* isolates [8]. Here, four typing methods (MLST, single-copy core genes phylogenetic analysis (SCCGPA), dispensable genes content pattern analysis (DGCPA) and CVTree) were compared using 23 antiserum standard *B. thuringiensis* strains from *aizawai*, *kurstaki*, *israelensis*, *thuringiensis* and *morrisoni* serovars. The CVTree was selected to evaluate the subtyping of 182 antiserum standard *B. thuringiensis* strains from 86 different serovars. The analysis was then extended to 2359 *B. cereus s.l.* strains. Finally, a public user-friendly web server—the *Bacillus* Typing Bioinformatics Database (BTBIDB)—which uses CVTree that would allow users to analyze the phylogenetic relationship of their strains with those in our database, was built. This study resulted in the description of an operational typing technique that shows a high level of confidence and that will be useful to assist in the future genomic analysis of *B. thuringiensis* strains and other closely related *B. cereus s.l.* species.

## 2. Results

### 2.1. Evaluation of Different Phylogenetic Analysis Methods for Typing B. thuringiensis Strains

**Figure 1:**
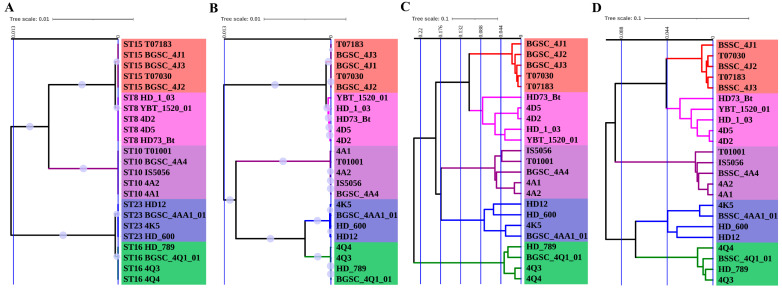
Phylogenetic analysis of 23 *B. thuringiensis* strains based on four typing methods. (**A**), multi-locus sequence typing (MLST); (**B**), single-copy core genes phylogenetic analysis (SCCGPA); (**C**), dispensable genes content pattern analysis (DGCPA); (**D**), composition vector tree (CVTree). Bootstrap support values were calculated from 1000 replicates for MLST and SCCGPA, but they were not applied for DGCPA and CVTree. Bootstrap values over 0.5 are indicated by filled purple circles on the branch. Colors of the branches and strain label background represent different serovars, red for *aizawai*, magenta for *kurstaki*, purple for *thuringiensis*, blue for *morrisoni* and green for *israelensis*. The blue vertical bars indicate the dissimilarity coefficient.

In this study, the unweighted pair group method with arithmetic mean (UPGMA) phylogenetic analysis was applied to 23 antiserum standard *B. thuringiensis* strains from five different serovars, and the convenience and the efficiency of four different typing approaches, MLST, SCCGPA, DGCPA and CVTree, were compared. First, the strain typing resolutions of the four methods were compared. Although antigen serotyping represents a crude typing system, all four genomic methods roughly agreed with antigen serotyping, revealing five main clusters corresponding to the different serovars (Figure 1). From this analysis, it was clear that the CVTree and DGCPA methods had a greater discriminatory potential than MLST and SCCGPA did, since the distances among individual strains was larger using these former typing methods.

Then, the efficiency of three genome-based methods, SCCGPA, DGCPA and CVTree, were evaluated from the perspective of time and resource consumption. Since all three methods used genomes as input data, we only compared the elapsed time in three dry lab steps, i.e., preparing the input data, calculating the dissimilarity matrix and inferring a phylogenetic tree based on the dissimilarity matrix. In the input data preparation step, CVTree directly used amino acid sequence data; so, no more processing was needed. Both SCCGPA and DGCPA needed 60,300 s to run the blast-all program (integrated into the PGAP software: version 1.2.1; Beijing Institute of Genomics, Chinese Academy of Sciences; Beijing, China) to recognize and extract the required data from genomes. In this study, 3653 single core gene clusters and 5626 dispensable gene clusters were recognized from the 23 genomes.

For the dissimilarity matrix calculation step, CVTree took 45 s. For SCCGPA, the MAFFT program took 4020 s to produce a multiple alignment file, and PHYLIP took 4800 s to produce the dissimilarity matrix; then, trimAI took less than a second to trim and produce the multiple alignment file. For DGCPA, an in-house Perl script took five seconds to produce a dissimilarity matrix.

Finally, for all methods just two seconds were required to build the trees.

In total, CVTree took 47 s to complete the process, while DGCPA and SCCGPA took 16.75 and 19.20 h, respectively (Table 1), showing that traditional sequence alignment-based methods are time-consuming. CVTree avoids sequence alignment by using k-string counts of all protein products encoded in a genome, and this alignment-free methodology was significantly faster than the other methods were.

### 2.2. CVTree Method for Bacillus thuringiensis Strain Typing

**Figure 2:**
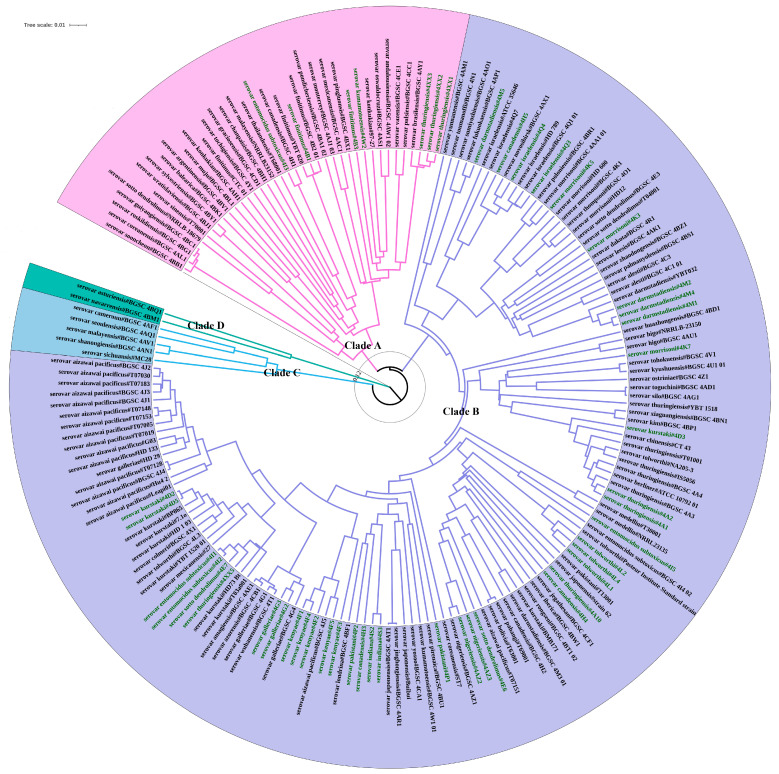
Phylogenetic analysis of 182 antiserum standard *B. thuringiensis* strains using CVTree. This tree was obtained using CVTree with k-string = 6 and PHYLIP. Colors of strain label backgrounds represent different clades. Strains with their names in green represent 47 newly sequenced BGSC *B. thuringiensis* strains.

A total of 182 antiserum standard *B. thuringiensis* strains from 86 different serovars were used as a model to evaluate the subtyping ability of CVTree to distinguish between isolates of the same species and to better assess the relationship between the genome sequence and serovar (Figure 2). Forty-seven strains from 16 serovars were sequenced by the staff in our laboratory, and the remaining one hundred and thirty-five strains from 84 serovars were obtained from the NCBI GenBank database. Details about each of the 47 newly sequenced genomes are provided in Table S1, which shows the genome size (base pairs), the predicted number of genes, CDS and tRNAs. These 47 genomes varied in size, ranging from 5.12–6.79 Mb, with the number of genes ranging from 5276–7121, indicating substantial genomic diversity within the newly sequenced *B. thuringiensis* strains.

The CVTree analysis of these 182 *B. thuringiensis* strains identified four major Clades (Clade A–Clade D) with dissimilarity coefficients values between 0.114–0.128 (Figure 2). These four Clades are in accordance with the distribution previously described by Zheng et al. on the basis of single-copy core genes sequence similarity [33]. The most common serovars were assigned to the largest Clade, B, including all strains previously classified as *kurstaki*, *israelensis*, *aizawai*, *morrisoni*, *darmstadiensis*, *tolworthi*, *kenyae* and *galleriae* serovars. Clade B also included most of the strains belonging to the *thuringiensis* (75%, n = 12), *canadensis* (80%, n = 5), *entomocidus* (80%, n = 5) and *sotto* (80%, n = 5) serovars. Clade B is considered to be the “Thuringiensis” [33] or “B. kurstaki and Bc” [18] clade. All five *B. thuringiensis* serovar *finitimus* strains were assigned to Clade A. Clades C and D represent two small clades containing some unique serovars (*cameroun*, *seoulensis*, *malayensis*, *shanongiensis* and *sichuansis* for Clade C, and *asturiensis* and *navarrensis* for Clade D). In most cases, strains with the same serovar were assigned into the same Clade, often to the same branch; yet, there are some ambiguous strains assigned to different positions of the tree, indicating either higher variation within these strains or incorrect serovar typing [15].

### 2.3. CVTree Method for Differentiating Bacillus thuringiensis from Other Closely Related B. cereus s.l. Species

To establish if our CVTree analysis correlates with Carroll et al.’s analysis, genomic information about 2359 strains belonging to 20 former *B. cereus s.l.* species were obtained from GenBank to perform this comparison (Table S2). These 2359 strains included 2055 strains used in study of Carroll et al. A high level of correlation was observed between CVTree and the ANI-based method, revealing 12 robust and well-separated genomospecies clusters. In addition, six putative novel genomospecies (from xiii to xviii) classified by Carroll et al. were also identified in our CVTree analysis (Figure S1). In the case of the novel genomospecies, xiv (identified as Unknown Species 14 by Carroll et al.), it was found that all nine strains in this group clustered with *B. luti* genomospecies. The ANI value between these nine strains and the closest *B. luti*-type strain, TD41, ranged from 91.3056 to 92.0269.

**Figure 3:**
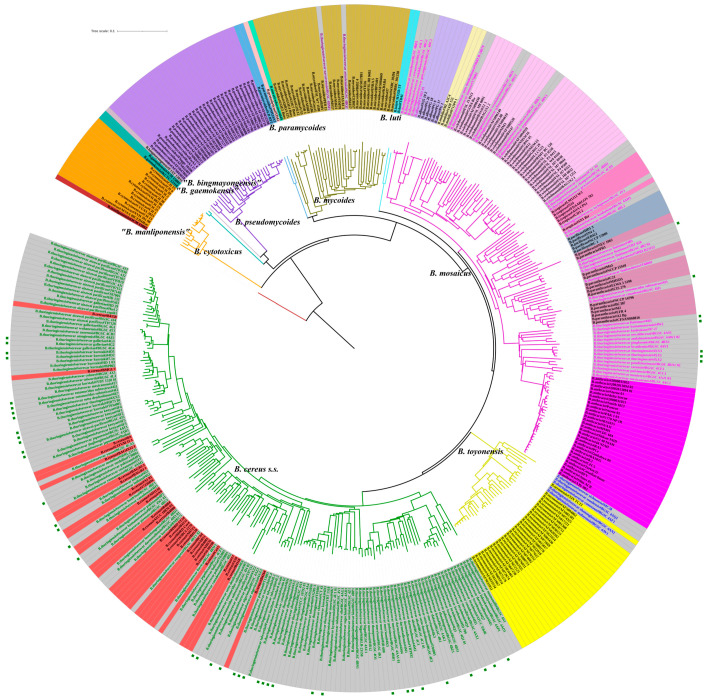
Phylogenetic analysis of 421 *B. cereus s.l.* strains using CVTree. This tree was obtained using CVTree with k-string = 6. Colors of strain label backgrounds represent previously designated species names. Colors of branches represent different clusters based on CVTree analysis where appropriate are labelled with the names of previously designated genomospecies classified by Carroll et al. For *B. thuringiensis* strains (grey background), the color of the text reflects the four primary clades identified in Figure 2: magenta for Clade A, green for Clade B, blue for Clade C and purple for Clade D. The outermost green squares represent the 47 newly sequenced BGSC *B. thuringiensis* strains. *B. manliponensis*#JCM_15802 was treated as an outgroup for which phylogeny was rooted.

To determine the classification ability of CVTree, 239 *B. cereus s.l.* strains, whose former species definitions were consistent with the genomospecies definitions, were selected and combined with the 182 antiserum standard *B. thuringiensis* strains analyzed above (Figure 3). Colored strain names on a grey background reflect the four primary clade distribution of 182 *B. thuringiensis* strains discussed above (magenta for Clade A, green for Clade B, blue for Clade C and purple for Clade D). All strains in Clade A, the “Anthracis” Clade clustered with the *B. mosaicus* genomospecies. Within this clade, fifteen *B. thuringiensis* strains clustered with *B. anthracis*, seven clustered with *B. paranthracis*, two clustered with *B. pacificus*, four clustered with *B. tropicus*, six clustered with *B. wiedmannii* and four clustered with *B. albus*. All the strains in Clade B, the “Thuringiensis” Clade, clustered with *B. cereus s.s.* genomospecies. All five strains in Clade C clustered with *B. toyonensis* genomospecies. Two Clade D strains clustered with *B. mycoides* genomospecies. Among the 47 newly sequenced *B. thuringiensis* strains, 7 clustered with *B. mosaicus* genomospecies, and the remaining 40 clustered with *B. cereus s.s.*

### 2.4. BTBIDB Web Server for B. cereus s.l. Phylogeny

The author of CVTree constructed an effective web server, CVTree 4 (http://cvtree.online/v4/prok/, accessed on 25 August 2021) [34], which is capable of comparing the tree branching orders using systematics at all taxonomic ranks from domains down to species and strains. However, this web server is focused on studies of phylogeny and taxonomy on a large scale. In this work, in order to facilitate the particular phylogeny study of *B. cereus s.l.* strains via CVTree, a free web server (BTBIDB, *Bacillus* Typing Bioinformatics Database, https://btbidb.com, accessed on 15 September 2021) was built. This web server possesses a comprehensive database of 4216 genomes from 136 *Bacillus* species (Table S3), including 2359 *B. cereus s.l.* strains and the 47 newly sequenced BGSC *B. thuringiensis* strains analyzed above. Via the “Testing Your Strain” section of BTBIDB, a logged-in user can quickly analyze the taxonomic placement of their own strains among selected genomes from the server’s inbuilt database. A detailed user manual for the web server can be found in the “Tutorial” section of BTBIDB.

## 3. Discussion

Horizontal gene transfer within the *B. cereus* group has been previously shown [18,33] and complicates the taxonomic classification of these bacteria, which has traditionally been defined by their phenotypic characteristics encoded in plasmid DNA. In particular, *B. thuringiensis* is defined by the possession of horizontally mobile genes encoding pesticidal proteins that are responsible for the production of crystalline inclusions within bacteria. However, several strains designated as *B. thuringiensis* neither carry insecticidal genes, nor produce visible crystals [33]. For example, 4H1, 4AY1 and 4BA1 strains, which lack crystal inclusions, were located in Clade A via the CVTree analysis in this work and were assigned to *B. mosaicus* genomospecies by Carroll et al., suggesting the incorrect classification of these strains. It is still challenging to distinguish between *B. thuringiensis* and *B. cereus s.s.* (Figure 3 and Figure S1), as others have observed [35].

The authors of several recent studies tried to solve the species–phenotype incongruences observed in the distribution of *B. thuringiensis* and *B. cereus* groups using genome-based taxonomic frameworks [24,25,36]. Via an adjustment of the ANI genomospecies threshold, Carroll et al. proposed an explicit, standardized framework for the taxonomic classification of *B. cereus* group, which accounted for both phylogenomic diversity and phenotypic heterogeneities. Using the SCCGPA approach, Zheng et al. analyzed the population structure of *B. cereus* group and found that most of *B. thuringiensis* strains were clustered into two main clades, with Clade 1 being the “Anthracis” clade and Clade 2 being the “Thuringiensis” clade. It is interesting to compare our CVTree result with the works of Zheng et al. and Carroll et al. A high degree of similarity was observed when the three output trees were compared. Exactly the same strains that we labeled as members of Clades C and D were included in Clades 3 and 4 in Zheng et al.’s work. In our CVTree, Clade A was subdivided into two main branches, which corresponded to Clade 1.1 and Clade 1.2 in Zheng et al.’s work, with the same strains grouped in these branches. Additionally, all strains in Clade A, the “Anthracis” Clade, clustered with the *B. mosaicus* genomospecies defined in Carroll et al.’s work. Regarding Clade B, our analysis showed that this is subdivided into two main branches, with one of them representing the strains that Zheng et al. cataloged as Clade 2.2. The other branch of our Clade B tree was subdivided into two branches that corresponded to two independent Clades, 2.1 and 2.3, in Zheng et al.’s work. All the strains in Clade B, the “Thuringiensis” clade, clustered with *B. cereus s.s.* genomospecies, as defined in Carroll et al.’s work. Additionally, we also found that *B. thuringiensis* strains belonging to Clade 2.3 in Zheng et al.’s work and were clustered with *B. cereus s.s.* strains, and strains belonging to Clades 2.1 and 2.2 formed standalone *B. thuringiensis* Clades lacking any *B. cereus s.s.* strains (Figure 3). These data confirm that CVTree is a method that gives results comparable to those of SCCGPA and ANI.

On a larger scale, the *B. cereus* group, also called the ‘Cereus clade’, and the ‘Subtilis clade’ together form two large distinct Clades of the genus *Bacillus*. Except for these two Clades, the *Bacillus* genus also contained many other divergent species, which exhibit extensive polyphyly and share very little in common with one another. By reconstructing multiple genomic-scale phylogenetic trees, Gupta et al. identified different monophyletic Clades of *Bacillus* species and a number of molecular markers that are specific and distinguishing characteristics of different *Bacillus* species Clades [37]. They found that in addition to the Cereus and Subtilis Clades, most of the *Bacillus* species consistently formed novel distinct Clades, which should be recognized as novel genera. Thus, in this study, we also compared our CVTree output with that of Gupta et al. This comparison is not intended to contribute to the debate about the classification of these *Bacillus* species, but rather to verify the typing ability of CVTree on a larger scale. Once again, CVTree has accurately revealed two main monophyletic Clades, ‘Cereus clade’ and ‘Subtilis clade’, as well as other novel distinct Clades proposed by Gupta et al. As shown in Figure S2, CVTree strongly supported the groupings of the Cereus Clade, which is grouped based on the phylogenetic and molecular distinctness in Gupta et al., including the ‘Core Cereus Clade’, and the other, more branching Panaciterrae and Luciferensis Clades. The novel species, *B. rhizoplanae* [38], was also grouped into the ‘Core Cereus Clade’.

Methodologically, genomic DNA sequences provide a number of ways to distinguish between different strains, including the analysis of core gene similarities (SCCGPA), the presence or absence of dispensable genes (DGCPA) [39] and the overall similarity between two genome sequences (ANI) [40]. For SCCGPA and DGCPA, both methods sample an enormously greater proportion of the genome than MLST does, but they still do not use whole-genome data. Among the initial 23 antiserum standard *B. thuringiensis* genomes analyzed in this work, core genes and dispensable genes account for 59.38–69.09% and 29.31–38.18% of the total number of genes, respectively. By contrast, both ANI and CVTree use whole-genome information. CVTree differs from the ANI method in that it uses all identified protein-encoding genes, and in terms of methodology it is an alignment-free method based on K-length peptide counting. The alignment-free methodology makes using CVTree significantly faster than using other methods is. Moreover, although the length of the peptide can be varied for prokaryotes, a value of five or six is recommended [41]. In this study, it was found that a value of 6 gave results consistent with previous phylogenetic analyses, and k-string = 6 was set during CVTree analysis.

## 4. Conclusions

In summary, the CVTree approach proved to be a quick and reliable method for determining the relationships between *B. thuringiensis* and other closely related *B. cereus s.l.* species. It provides data that are consistent with other genomic phylogeny approaches. More importantly a free, web-based tool—the *Bacillus* Typing Bioinformatics Database (https://btbidb.com, accessed on 15 September 2021)—which allows users to compare their own strains against a comprehensive database, was built. This server will be useful for researchers with a specific interest in taxonomy or to help other researchers interested in the characterization of risk assessment of specific strains within this group.

## 5. Materials and Methods

### 5.1. Bacterial Strains and Data Resources

In this work, genomic data from 4216 strains belonging to 136 different *Bacillus* species, including 2359 *B. cereus s.l.* strains, were collected (Tables S2 and S3). These included 2359 *B. cereus s.l.* strains, such as 955 *B. cereus s.s.*, 540 *B. thuringiensis*, 259 *B. anthracis*, 214 *B. toyonensis*, 154 *B. wiedmannii*, 110 *B. pseudomycoides*, 67 *B. mycoides*, 14 *B. cytotoxicus*, 14 *B. paranthracis*, 7 *B. albus*, 6 *B. pacificus*, 6 *B. tropicus*, 3 *B. mobilis*, 2 *B. paramycoides*, 2 *B. luti*, 2 *B. gaemokensis*, 1 *B. manliponensis*, 1 *B. bingmayongensis*, 1 *B. proteolyticus* and 1 *B. nitratireducens*. Except for forty-seven *B. thuringiensis* strains newly sequenced in our laboratory, as described below, the rest were collected from the NCBI GenBank database (including finished annotated genomes and assemblies, ftp://ftp.ncbi.nlm.nih.gov/genomes/genbank/bacteria/, accessed on 15 January 2021). These strains were named by combining their species types, serovar types and original strain names, such as *B. thuringiensis*#serovar_kurstaki#BMB171. One *B. weihenstephanensis* strain (*B. mycoides*#WSBC_10204) was assigned as *B. mycoides* species in the NCBI GeneBank Database. Because two species were reported to be closely related, this assignment was not changed here. QUAST [42] was performed to evaluate genome assemblies via computing the number of contigs, largest contig length, GC content and N50 length (Table S3).

### 5.2. Genome Sequencing and Assembly

For genome sequencing, strains were grown overnight on LB agar plates. The cells were harvested, and 100 mg of Bt cells was washed with ddH_2_O, and their DNA was extracted as previously described [43]. DNA samples were stored at −20 °C until sequencing. Sequencing was performed using an Illumina HiSeq 2500 sequencer (Illumina, San Diego, CA, USA). The reads were cleaned by removing those with Ns or more than 20% low-quality bases, and 1 Gb 2 × 100 bp pair-end clean reads for each strain were obtained. SPAdes (version 3.13.1) [44] with default settings was employed for genome assembly. Genome assemblies were then submitted to the NCBI database for genome annotation via the NCBI Prokaryotic Genome Annotation Pipeline [45].

### 5.3. Evaluation of Phylogenetic Analysis Methods

In order to evaluate the convenience and efficiency of different approaches for the phylogenetic analysis of strains, 23 *B. thuringiensis* strains that belonged to five common serovars (*aizawai*, *kurstaki*, *israelensis*, *thuringiensis* and *morrisoni*) were randomly selected to perform MLST, SCCGPA, DGCPA and CVTree analyses. All dry lab analyses were performed using an LENOVO x3850 X6-[6241S1C]-/00FN846 server running CentOS Linux release 7.5.1804, with 4 × 2.00 GHz Intel(R) Xeon(R) processors and 16 GB of 1600 Mhz DDR3 RAM, and one thread was used.

Input data were prepared as follows: For the MLST approach, seven housekeeping gene sequences (*glpF*, *gmK*, *ilvD*, *pta*, *pur*, *pycA* and *tpi*) from the selected 23 *B. thuringiensis* strains were identified by nucleotide sequencing based on the polymerase chain reaction (PCR) [15] or extracted from genome sequences using BTyper (version 2.3.3) [46]. For SCCGPA and DGCPA, the pan-genome analysis pipeline (PGAP version 1.2.1) [47] was employed to identify core and dispensable genes using an identity threshold of 50%; core genes were defined as those present in all strains, and dispensable genes were those shared partially among strains [48]. Finally, for CVTree amino acid sequence data were directly used. The same annotated genomes with clearly described coding sequences (CDS) of proteins were used.

Dissimilarity matrices were calculated as follows: For MLST and SCCGPA, the nucleotide sequences of selected genes were concatenated, and then multiple alignments were performed using MAFFT (version 7.427) [49]. TrimAI (version 1.2rev59) [50] was used to remove the poorly aligned regions of multiple sequence alignments and produce a multiple alignment file in PHYLIP format for subsequence analysis. The dissimilarity matrix between strains was counted using SEQBOOT and DNAdist (PROTdist for protein sequence) routines within PHYLIP (version 3.698) [51]. Bootstrap tests in the MLST and SCCGPA analyses were performed with 1000 replicates. For DGCPA analysis, the dispensable gene profile was recorded based on whether a particular locus was present (scored as 1) or absent (scored as 0) in the genome. The dissimilarity matrix between strains was counted based on the difference of each locus using an in-house Perl script. Finally, for CVTree analysis, the dissimilarity matrix between strains was calculated based on the CV types of each strain using the CVTree standalone version, with k-string = 6 [41].

For phylogenetic analysis, UPGMA is a simple agglomerative hierarchical clustering method used to produce a dendrogram from a dissimilarity matrix, assuming that the distances from the root to each branch tip are equal [52]. With an orderly branch length, this tree provides a describable and quantifiable evaluation method of strain typing, and the evolutionary distances between strains are represented as the values of their dissimilarity coefficients, corresponding to the tree scale, where a larger dissimilarity coefficient indicates a greater distance between strains. In this study, UPGMA phylogenetic trees were constructed based of the dissimilarity matrix using the NEIGHBOR program of PHYLIP (version 3.698) [51]. All tree files were annotated using an online tool, iTOL (Interactive Tree of Life, https://itol.embl.de/, accessed on 18 March 2021) [53].

## Figures and Tables

**Table 1:** Time consumption comparisons of three methods of genome comparison.

Method	Run Time
CVTree	47 s
DGCPA	16.75 h
SCCGPA	19.20 h

## Data Availability

Genome sequences of all strains used in this study were available in GenBank under the accession numbers shown in Tables S1–S3.

## Supplementary Materials

The following supporting information can be downloaded at: https://www.mdpi.com/article/10.3390/toxins15060393/s1. Table S1: 182 *B. thuringiensis* strains used in this study; Table S2: 2359 *B. cereus s.l.* strains used in this study; Figure S1: Phylogenetic analysis of 2359 *B. cereus s.l.* strains using CVTree. This tree was obtained using CVTree with k-string = 6. Colors of strain label backgrounds represent previously designated species names. Colors of branches represent different clusters based on the CVTree analysis. Colors of the outermost circle represent different genomospecies classified by Carroll et al., with white representing strains not included in that study. *B. manliponensis*#JCM_15802 was treated as an outgroup on which the phylogeny was rooted; Table S3: 4216 *B. cereus s.l.* built-in strains in BTBIDB web server; Figure S2: Phylogenetic analysis of *Bacillaceae* species, including all named *Bacillus* species and species from related genera, using CVTree. This tree was obtained using CVTree with k-string = 6. Except for Cereus Clade and Subtilis Clade, species from other *Bacillaceae* clades/genera that were proposed by Gupta et al. are compressed. Non-validly described species in the tree are shown within quotation marks: ‘species name’.
